# Supplementary material for: Controlled Hydrothermal Growth and Li+ Storage Performance of 1D VOx Nanobelts with Variable Vanadium Valence
Source: Nanomaterials (Basel). 2019 Apr 17;9(4):624. doi: 10.3390/nano9040624 (PMC6523597; doi:10.3390/nano9040624)
Supplement: Supplementary file 1 [file nanomaterials-09-00624-s001.zip › nanomaterials-484184-SI-proof done.pdf]

Supplementary Materials: Controlled Hydrothermal Growth and Li+ Storage Performance of 1D VO_x Nanobelts with Variable Vanadium Valence

Yuhan Jiang, Xiaowei Zhou *, Xu Chen, Jia Wen, Linlin Guan, Mingxia Shi, Yang Ren and Zhu Liu *

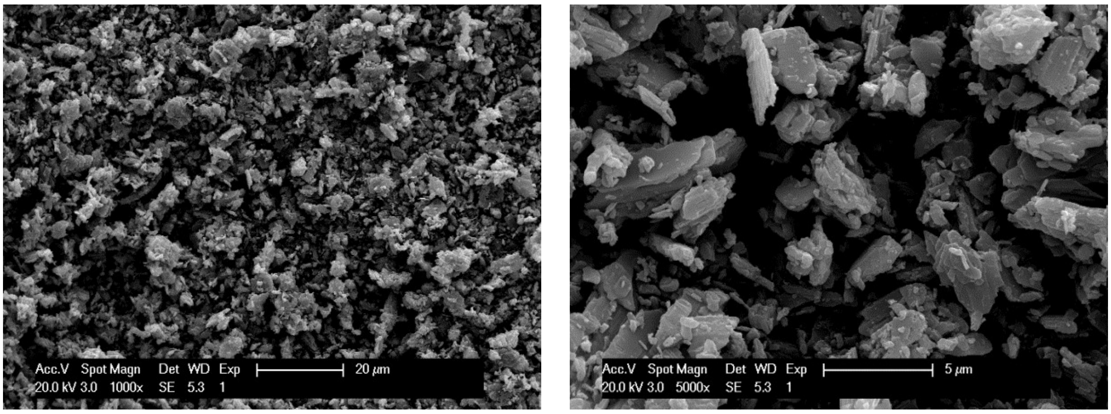

(**a**) (**b**)

Figure 1. SEM images of commercial V_2O_5 powder at 1000 (**a**) and 5000 (**b**) times magnification, respectively.

The Figure S1 above is the SEM images of commercial V_2O_5 powder at different magnifications. We can see that the size distribution of V_2O_5 particles is not uniform and their diameter is in the range from hundreds of nanometers to a dozen of micrometers. The shape of these V_2O_5 particles is also irregular. V_2O_5 itself has low solubility in water, but it will form the metastable vanadium oxide precursors, which disperse well, in the special high temperature and pressure hydrothermal environment. Then, the oriented crystallization growth of VO_x happens, leading to the formation of one-dimensional nano-belted structure.

Reducing action of ethanol (EtOH) under hydrothermal condition: The hydrothermal reaction itself could provide a reductive atmosphere [1]. EtOH can behave as a kind of weak reducing agent in the hydrothermal environment. Some reports on the use of ethanol as a reducing agent have been reported [2,3]. EtOH will be oxidized and converted to aldehydes and at the same time vanadium element would be reduced to the lower valence state [Possible reaction equation can be roughly written as $(5-2x)CH_3CH_2OH + V_2O_5 \rightarrow 2VO_x + (5-2x)CH_3CHO + (5-2x)H_2O$ $(2 < x < 2.5)$].

The inductive reducing action of MWCNTS: MWCNTs are not well dispersed in water and it only shows the temporary state of suspension in solution. However, under the special high-temperature and pressure liquid environment of hydrothermal reaction, MWCNTs can be dispersed evenly to ensure their sufficient contact with vanadium oxide precursors. Because the MWCNTs here used were previously treated with mixed concentrated acids in order to get better dispersion in liquid condition. Generally, the mixed concentrated acids are H_2SO_4 (>70 wt%) and HNO_3 (~65 wt%) according to 3:1 (volume ratio). The acid treatment process will create some surface hydroxyl, carbonyl and carboxyl functional groups [4,5]. These functional groups on MWCNTs contribute to the further reduction of vanadium oxide precursors under the hydrothermal condition and the presence of EtOH.

The possible growth mechanism of vanadium oxide nanobelt: Under the special high temperature and high pressure hydrothermal condition, the commercial V_2O_5 powder will disperse to form the metastable VO_x precursor. Owing to the role of energy modulation under hydrothermal condition, the growth kinetics of vanadium oxide precursor is faster in a specific direction during the process of self-assembly crystallization and conversely the growth in other orientations are inhibited, resulting in a one-dimensional VO_x nano-belted morphology. As some literatures reported

[6,7], we know that hydrothermal reaction is a common method that can be adopted to prepare a series of one-dimensional metal oxide nanomaterials.

In the post-sintering of VO_x NBs in air, the V valence state will increase by in-situ oxidation, but the 1D morphology of VO_x NBs could be maintained and form V_2O_5 NBs with high V valence state.

References:

1. Huang, H.-H.; De Silva, K.K.H.; Kumara, G.R.A.; Yoshimura, M. Structural Evolution of Hydrothermally Derived Reduced Graphene Oxide. *Sci. Rep.* **2018**. *8*, 6849.
2. He, H.; Li, Y.; Zhang, X.;Yu, Y.; Zhang, C. Precipitable silver compound catalysts for the selective catalytic reduction of NOx by ethanol. *Appl. Catal. A Gen.* **2010**, *375*, 258–264.
3. Sylvai, B.; Cheloha, R.W.; Berkowitz, D.B. Enantioselective, Ketoreductase-Based Entry into Pharmaceutical Building Blocks: Ethanol as Tunable Nicotinamide Reductant. *Org. Lett.* **2009**, *11*, 305–308.
4. Yue, L.; Li, W.; Sun, F.;Zhao, L.; Xing, L. Highly hydroxylated carbon fibres as electrode materials of all-vanadium redox flow battery. *Carbon* **2010**, *48*, 3079–3090.
5. Zhang, L.; Hashimoto, Y.; Taishi, T.; Ni, Q.-Q. Mild hydrothermal treatment to prepare highly dispersed multi-walled carbon nanotubes. *Appl. Surf. Sci.* **2011**, *257*, 1845–1849.
6. Liu, H.D.; Hu, Z.L.; Su, Y.Y.; Hu, R.; Tian, L.L.; Zhang, L.; Ruan, H.B. Facile Preparation of 1D α-MnO_2 as Anode Materials for Li-ion Batteries. *Int. J. Electrochem. Sci.* **2016**, *11*, 8964–8971.
7. Xing, X.; Cai, Y.; Chen, N.; Li, Y.; Deng, D.; Wang, Y. Synthesis of mixed Mn–Ce–O_x one dimensional nanostructures and their catalytic activity for CO oxidation. *Ceram. Int.* **2015**, *41*, 4675–4682.
